# Long-term survival in metastatic malignant struma ovarii treated with oral chemotherapy: A case report

**DOI:** 10.3892/ol.2014.2587

**Published:** 2014-10-03

**Authors:** MASAYO UKITA, HIDEKATSU NAKAI, YASUSHI KOTANI, TAKAKO TOBIUME, EIJI KOIKE, ISAO TSUJI, AYAKO SUZUKI, MASAKI MANDAI

**Affiliations:** Department of Obstetrics and Gynecology, Kinki University Faculty of Medicine, Osakasayama, Osaka 589-8511, Japan

**Keywords:** malignant struma ovarii, metastasis, long-term survival, chemotherapy

## Abstract

Malignant struma ovarii is a rare type of ovarian tumor. Metastasis from malignant struma ovarii is rare and has only been documented in 5–6% of cases. The natural history and optimal treatment strategy for malignant struma ovarii remains controversial due to its rarity. The current report presents the case of a 45-year-old female who presented with a tumor of the rib bone. Following resection, the postoperative diagnosis was a metastasizing thyroid carcinoma. No abnormality was detected in the thyroid gland, however, computed tomography revealed a tumor in the left ovary. The patient underwent a left salpingo-oophorectomy and a wedge resection of the right ovary. The postoperative diagnosis was determined as a mature cystic teratoma with malignant struma ovarii (thyroid type, follicular carcinoma) of the left ovary and mature cystic teratoma of the right ovary. Four years subsequent to the initial diagnosis, multiple lung metastases were detected. The following chemotherapies were administered sequentially and intermittently: Tegafur-uracil, paclitaxel/carboplatin and oral etoposide. During this period, the metastatic lesions extended into the bone and progressed slowly. The patient continues to survive with the disease and 24 years have passed since the initial diagnosis, 20 years following the diagnosis of multiple lung metastates. The present report describes a rare case of malignant struma ovarii in which surgical resection and pathological examination of a metastatic rib tumor resulted in the identification of the primary ovarian lesion. The clinical behavior of malignant struma ovarii does not necessarily indicate a histological malignancy, therefore, prediction of future metastasis is difficult and the optimal treatment strategy for malignant struma ovarii is controversial. The present case indicates that the long-term use of oral anticancer agents may facilitate the maintenance of tumor dormancy.

## Introduction

Struma ovarii is a rare form of ovarian germ cell tumor and was first described by Gottschalk in 1899 (1). Although ~5–15% of teratomas contain thyroid tissue, thyroid tissue must be predominant in the teratoma for the cancer to be classified as a struma ovarii (2). Struma ovarii comprise only 1.4–2.7% of teratomas and only 1% of ovarian tumors, worldwide (1,3,4). Malignant struma ovarii is even less common and its associated metastasis is documented in <5–6% of malignant struma ovarii cases (2,5,6). The natural history and optimal treatment strategy for malignant struma ovarii remains controversial due to its rarity. Previous reports have proposed that the patient should undergo complete surgical staging for ovarian cancer, with other reports indicating that a total thyroidectomy and adjuvant ^131^I radioablation therapy may aid in the elimination of residual thyroid tissue following surgical removal of the primary tumor (2). The current report presents a case of long-term survival of malignant struma ovarii with multiple lung and bone metastases by treatment with chemotherapy. Written informed consent was obtained from the patient.

## Case report

**Figure 1:**
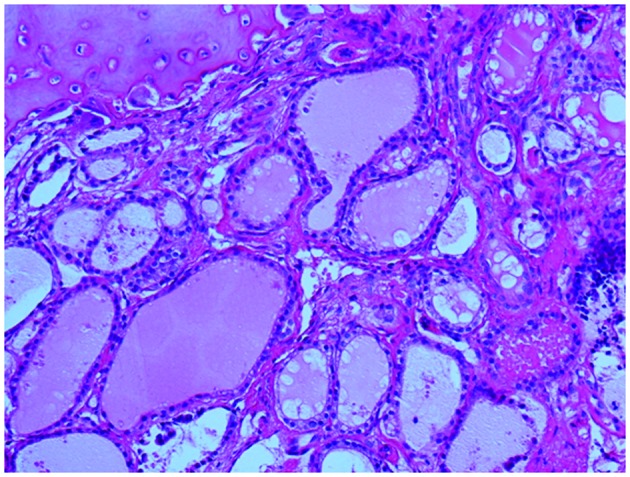
Microscopic view of the bone metastasis in a tissue sample taken during the primary surgery. Folicular structures which contain the colloid proliferate invasively via destruction of the trabeculae, indicating a diagnosis of metastatic thyroid carcinoma (hematoxylin and eosin stain; magnification, ×200).

In October1989, a 45-year-old premenopausal female (gravida 2; para 2) was referred to the Department of Obstetrics and Gynecology, Kinki University Hospital (Osaka, Japan) for further examination of a metastatic bone tumor. In September 1989, the patient had consulted the local doctor due to back pain and a tumor was identified in the right ninth rib. The patient was admitted to the Department of Orthopedics (Kinki University Hospital) and underwent tumor resection. Pathological diagnosis of the surgical specimen revealed folicular structures which contain the colloid had proliferated invasively via the destruction of the bone trabeculae and metastasis was suspected, which resulted in a diagnosis of a metastasizing thyroid carcinoma (Fig. 1). An ultrasound examination of the thyroid gland and an evaluation of the thyroid hormonal function did not present any unusual findings. Computed tomography (CT) was then performed, which identified an ovarian tumor. The patient was subsequently referred to the Department of Obstetrics and Gynecology (Kinki University Hospital). Abdominal ultrasonography revealed a hypoechoic mass in the left ovary and magnetic resonance imaging revealed a 12×9.5×11-cm solid cystic mass with fatty tissue elements and calcification. In January 1990, a left salpingo-oophorectomy and a wedge resection of the right ovary were performed. Intraoperative examination revealed that the left ovary (diameter, 12 cm) was elastic and comprised of partially multilocular lesions and the right ovary (diameter, 4 cm) contained ovarian cysts. Gross examination of the uterus revealed that it was normal, however, a small volume of ascites was detected.

**Figure 2:**
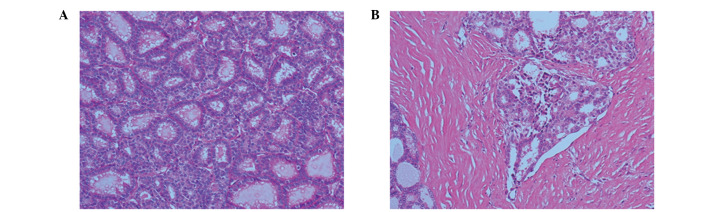
Microscopic view of the primary ovarian lesion. (A) Thyroid follicles of various size are densely proliferated. The follicular cells exhibit enlarged nuclei, chromatin condensation and nuclear stratification (hematoxylin and eosin [H&E] stain; magnification, ×200). (B) Vascular invasion is also apparent (H&E stain; magnification, ×100). The subsequent pathological diagnosis was malignant struma ovarii (thyroid type, follicular carcinoma).

The postoperative diagnosis was determined as a mature cystic teratoma with malignant struma ovarii (thyroid type, follicular carcinoma) of the left ovary and mature cystic teratoma of the right ovary. Microscopic analysis aided in the classification of the tumor as a follicular adenocarcinoma. The tumor cells formed tightly packed small and large follicles filled with pink colloid-like material, and exhibited round nuclei, increased chromatin (Fig. 2A), as well as mitoses and vascular invasion (Fig. 2B). Furthermore, ^123^I scintigraphy revealed no abnormal uptake in the thyroid or other organs. The patient was finally diagnosed with stage IV malignant struma ovarii with rib metastasis, according to the International Federation of Gynecology and Obstetrics ovarian cancer staging system, 1988 (7).

**Figure 3:**
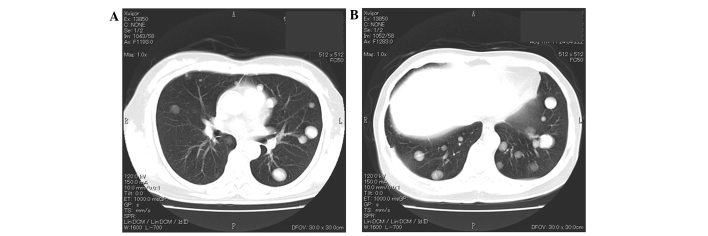
Computed tomography images of the (A) middle and (B) lower lung fields, prior to commencing paclitaxel/carboplatin therapy, indicating multiple metastases of the bilateral lung.

**Figure 4:**
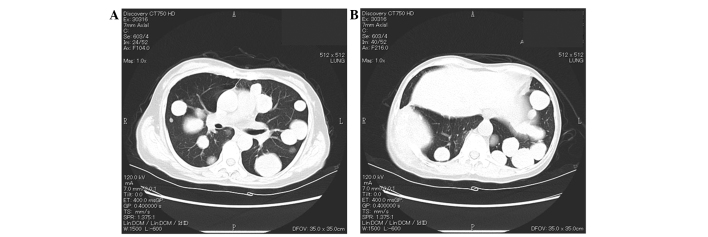
Computed tomography image (A) middle and (B) lower lung fields, eleven years after paclitaxel/carboplatin therapy, indicating slow growth of the metastatic lesions. No additional metastases was detected in any other organs.

Tegafur-uracil (UFT) was administered (600 mg/day) as an adjuvant chemotherapy for two years. Follow-up included measurements of thyroid function (via thyroglobulin levels) and regular chest X-rays every two months. In November 1993, three years after commencing UFT therapy, a chest X-ray revealed multiple lung metastases. Although systemic chemotherapy was recommended, the patient refused and recommenced treatment with UFT at an increased dosage of 3,000 mg/day. Following two further years of treatment, the patient refused to continue UFT administration, preferring to undergo observation. During this period, the lung nodules progressed slowly, however, no thyroid dysfunction or other symptoms were observed. In 1999, the patient identified a painless mass under the right scapula but did not recieve medical treatment. In 2001, the patient returned to Kinki University Hospital presenting with pain in the pubic region and a CT scan revealed metastasis to the left acetabulum. Paclitaxel (60 mg/m^2^) and carboplatin (area under the curve, 2) were administered weekly for six months resulting in stabilization of the lung nodules and the mass under the right scapula, however, the bone metastases in the acetabulum continued to progress. Following six months of paclitaxel/carboplatin therapy (Fig. 3), the patient refused any aggressive intervention and oral etoposide (25 mg/day) was administered intermittently for eleven years. After eleven years, due to the risk of secondary leukemia, oral etoposide was replaced with cyclophosphamide hydrate and continued until the present. During this period, the metastatic lesions progressed slowly (Fig. 4). Leg pain that is associated with the bone metastases is currently controlled by a non-steroidal anti-inflammatory medicine. Consequently, the patient has survived with the disease for 24 years since the initial diagnosis of stage IV malignant struma ovarii and for 20 years since the cancer recurred.

## Discussion

Malignant struma ovarii is a particularly rare type of tumor, which arises within mature teratomas in 0.1–0.3% of cases (2,6,8). According to the literature, metastasis from malignant struma ovarii has only been reported in 5–6% of cases (7). Robboy *et al* (9) estimated the annual incidence as <1 in 10,000,000 females per year in the USA. Continuous follow-up over a long period is considered to be necessary as in certain cases, particularly in the follicular type, the cancer may recur in subsequent years. In the present case, although the tumor was stage IV advanced, the primary tumor and the bone metastases were surgically resected and the patient remained tumor-free for four years until multiple lung metastases developed.

The physical presentation of malignant struma ovarii is non-specific. The majority of patients present with abdominal swelling, pain or a mass (2,9,10) and hyperthyroidism occurs in 8% of patients (8). Furthermore, the diagnosis of malignant struma ovarii is predominantly determined subsequent to surgical resection of the primary ovarian tumor (10,11). Thus, the present case is rare in its process of diagnosis, which was as follows: Initially the patient suffered from back pain and a rib tumor was identified; pathological examination of the resected tumor indicated a metastatic carcinoma originating from the thyroid, however, a thorough examination of the thyroid gland revealed no primary tumor; finally a subsequent CT scan identified an ovarian mass. The pathological findings of the ovarian tumor were consistent with a metastatic tumor, specifically a malignant struma ovarii. To the best of our knowledge, only one such similar case exists in the literature, in which metastasis to the femur preceded the diagnosis of a primary ovarian tumor. However, the clinical course of the case was not reported in detail (9). Although metastasis of malignant struma ovarii is rare, an ovarian origin should be considered when a metastatic thyroid tumor with no apparent thyroid origin is identified.

The diagnosis of malignant struma ovarii remains controversial. The pathological diagnosis is primarily established according to the criteria for tumors of the thyroid gland, which includes the presence of papillary formations lined with overlapping ground glass nuclei and vascular invasion. Papillary carcinoma is the most common type of malignant struma ovarii (10,11), however, histological malignancy in struma does not necessarily indicate a biological malignancy. Robboy *et al* (9) analyzed 88 cases of malignant struma ovarii (the largest series reported thus far) and classified the tumors into biological and histologic malignancies. A biological malignancy was defined as: i) The ovarian tumor having metastasized beyond the ovary, or having penetrated the ovarian surface; or ii) the ovarian tumor having recurred, regardless of earlier observations in the ovary. Of the 88 cases, 27 cases were classified as biologically malignant, however, of these only 12 cases were also classified as histologically malignant. The tissue from the remaining 15 cases was identified as histologically benign and unremarkable thyroid tissue. Histologically benign struma ovarii is also termed proliferative struma ovarii and is characterized by hyperplastic-type papillary formations and densely packed small follicles, however, it lacks the cytologic features required for a histologic malignancy. By contrast, among the 28 cases that were classified as histologically malignant, only 10 cases were identified as biologically malignant. Therefore, histological malignancy could not effectively be used to predict the subsequent clinical course. The 28 histologically malignant cases in the study by Robboy *et al* (9) consisted of 20 cases of papillary carcinoma, four cases of follicular carcinoma and four cases of a follicular variant of papillary carcinoma. Among these, 4 (20%), 4 (100%) and 2 (50%) cases, respectively, demonstrated biological malignancy. Furthermore, all four cases of follicular carcinoma exhibited extra-ovarian metastasis at the time of primary surgery. The present report, which demonstrated bone metastases at initial diagnosis, was classified as a follicular carcinoma, indicating that this histological subtype is more susceptible to early metastasis. Furthermore, in primary thyroid cancer, follicular carcinoma has a higher frequency of hematogenous metastasis when compared with papillary carcinoma (12).

The treatment of malignant struma ovarii remains controversial due to its rarity, as well as the difficulty in distinguishing between biologically benign and malignant cases (8). Surgical procedures range from conservative surgery, in which fertility is preserved, to staging surgery. The metastatic pattern of malignant struma ovarii resembles that of ovarian cancer. Metastasis can occur via the regional lymphatic system to the pelvic and paraaortic lymph nodes, directly to the omentum, the peritoneal cavity or the contralateral ovary, or hematogenously to the bone, lungs, liver and brain (2,5,6,8). Therefore, for advanced or disseminated disease, certain reports propose performing complete staging surgery for ovarian cancer, including pelvic and paraaortic lymph node sampling, peritoneal washing cytology, and omentectomy (2,10,11,13). Adjuvant therapy is not yet standardized, however, in previous reports ^131^I therapy, radiation and chemotherapy have been utilized (9). Vadmal *et al* (12) and Brenner *et al* (14) stated that in patients with residual abdominal disease, or with recurrent or metastatic tumors, a total cervical thyroidectomy followed by the administration of ^131^I therapy may be effective. DeSimone *et al* (2) reviewed 24 cases in the literature, in which no recurrence occurred in the four patients treated with adjuvant ^131^I therapy and seven of eight patients that were treated with ^131^I therapy following recurrence initially achieved a complete response. Thus, it was proposed that thyroidectomy and ^131^I therapy should be considered as the first line of management for malignant struma ovarii. One case report described chemotherapy as a treatment modality. Pardo-Mindan and Vazquez (15) administered L-phenylalanine mustard, Adriamycin and vincristine to a patient exhibiting extended dissemination, however, the tumor continued to grow. Chemotherapy was considered in the present case as malignant struma ovarii is classified as a malignant germ cell tumor. The administration of bleomycin was avoided due to the presence of multiple lung metastases and instead paclitaxel/carboplatin was administered, however, this treatment regimen was not effective. The patient declined aggressive intervention, therefore, oral anticancer agents were administered for long-term treatment. During this period, the tumor progressed slowly. Although it is not apparent in the present case, oral anticancer agents may be effective in maintaining tumor dormancy in slow-growing tumors.

In conclusion, the present report describes the case of a long-term survivor of metastatic malignant struma ovarii. Despite extra-ovarian metastasis at the initial diagnosis, the patient has survived with the disease for >20 years. Certain malignant struma ovarii are slow-growing and may have a long clinical course, therefore, aggressive cytotoxic chemotherapy may not be effective, although long-term use of an oral chemo-reagent may have certain benefits. These characteristics, as well as the possible efficacy of ^131^I therapy, indicate that malignant struma ovarii tumors resemble a primary thyroid carcinoma more than an ovarian germ cell tumor. Recent advances in molecular research of thyroid cancer have indicated the importance of activated MAPK and PI3K/AKT signaling pathways (14). Additionally, clinical trials with an anti-angiogenic reagent are ongoing (16). Future applications of these reagents may benefit patients presenting with malignant struma ovarii. Due to the rarity of malignant struma ovarii, it would be difficult to conduct a prospective trial to establish the ideal treatment; therefore, the development of biomarkers that distinguish between biologically malignant and benign tumors is required.
